# Socioeconomic Correlates and Key Aspects of Tobacco Surveillance Using Global Adult Tobacco Survey Among College Students of Mangaluru, South India

**DOI:** 10.7759/cureus.4115

**Published:** 2019-02-21

**Authors:** Praveen S Jodalli, Ganesh Shenoy Panchmal

**Affiliations:** 1 Public Health Dentistry, Yenepoya Dental College and Hospital, Mangaluru, IND

**Keywords:** tobacco use, smoking, smokeless tobacco, socioeconomic status

## Abstract

Introduction

Tobacco use in recent times has been identified to be the single biggest cause of morbidity and mortality. The epidemic of tobacco use has increased among young adults, which has changed the equation of the prevalence. The contribution of tobacco use to socioeconomic inequalities in health is increasing in India. Adolescent’s tobacco use may play an important role in increasing social inequalities related to smoking and smokeless tobacco use. The objective of this research was to study the association between socioeconomic status and tobacco use among college students of Mangaluru, South India

Methods

To analyze the association between the socioeconomic status and tobacco use, the study was conducted among 18 to 24-year-old college students (*n* = 802) in different colleges of Mangaluru, South India. A subset of key questions from the Global Adult Tobacco Survey (GATS) was used. The socioeconomic status of the participants was recorded using Kuppuswamy socioeconomic scale (for India) to categorize them into upper class and lower class. Descriptive statistics were applied to assess the factors related to tobacco use and socioeconomic status using SPSS ver. 24.0.

Results

Approximately 29.7% males and 70.3% of females completed the interview. Among 802 subjects, 69.9% belonged to the upper class and 30.04% belonged to the lower class. The current smokers who smoked daily 1.7% were from the upper class and 1.7% were from the lower class; no statistically significant difference was observed as well (*p *= 0.97). Approximately 3.4% from the upper class smoked less than daily and 3.1% from lower class smoked less than daily (NS). Among the upper class, 1.8% used daily and 2% subjects from the lower class used smokeless tobacco. A statistically significant difference was observed with subjects between the upper and lower class in noticing cigarette promotions in various forms during the last 30 days of interview.

Conclusion

Socioeconomic disparities on tobacco use need to be explored to ensure the initiation of new tobacco control activities and monitor the existing tobacco control policies. The current study finding demonstrates a significant but varied role of socioeconomic status on current and past tobacco use.

## Introduction

Tobacco use causes more than five million deaths annually and it is the leading preventable cause of death in the world [1-2]. By 2030, it is estimated that about 80% of deaths related to tobacco use will occur in middle-income and low-income counties [2]. A strong momentum at the global level against tobacco use is established by the Framework Convention on Tobacco Control through the World Health Organization (WHO) [3-5]. Overall, there is an increase in attention to the pattern of tobacco use in the low- and middle-income countries.

India is the third largest tobacco-producing nation and the second largest consumer of tobacco products. The Global Adult Tobacco Survey-2 (GATS-2) for India revealed that the prevalence of current tobacco use in any form among adults is 28.6% (266.8 million individuals) among adults aged 15 and above. From GATS-1 in 2009-2010 to GATS-2 in 2016-2017, the prevalence of any form of tobacco use has decreased significantly by 6% from 34.6% to 28.6%. The relative decrease in the prevalence of tobacco use is 17.3% [6].

The tobacco epidemic varies significantly from country to country. A systematic review and meta-analysis published in 2017 found a consistent inverse dose-response relationship between cigarette smoking and income level in the WHO regions for the United States of America, South East Asia, Europe, and Western Pacific [7]. In the low- and middle-income countries, it was found that the socioeconomic inequalities do exist in the pattern of tobacco use [8]. The pattern of tobacco use is inversely related to the socioeconomic status, with disadvantaged groups in the population being more likely to take up and continue smoking [9]. The exact mechanism behind the socioeconomic differences in smoking/smokeless tobacco use is unknown, but they have been linked to impoverished environments and lack of access to positive activities and alternatives to drugs [10].

Along with socioeconomic status of the families, young adult behaviors are influenced by their educational institutions. The socioeconomic environment where the school/college is located may influence the tobacco use pattern through several mechanisms, including exposure to tobacco advertisement, availability of tobacco products, and the development of social norms that facilitate youth smoking [11-13]. However, we could not find literature in regard to this in the developing countries like India that would address the impact of socioeconomic context of the colleges among students. The primary objective of the study was to find the relationship between socioeconomic status and the tobacco use among the students in the colleges of Mangaluru, south India.

## Materials and methods

This study was implemented to explore tobacco use among the population aged 18-24 years, men and women, attending colleges in Mangaluru. The tobacco use status and sociodemographic data were determined based on the questions from the GATS. A total of 802 subjects completed the interview. The GATS subset questionnaire consists of a core set of questions, which gathered information on the subject’s background characteristics, tobacco use (smoked or smokeless). The socioeconomic status of the participants was recorded using Kuppuswamy socioeconomic scale (for India); further, the study participants were categorized as upper class and lower class. The sample for the study was selected from the colleges using a multistage design to ensure that adequate coverage of the entire target population while simultaneously minimizing the data collection costs. The target population for this study included both men and women aged 18-24 years old who considered Mangaluru to be their primary place of residence, who consented to be part of the study. Our study excluded the institutionalized population and the subjects unwilling to consent.

Study variables

Information on tobacco use that included smoking and smokeless tobacco was recorded keeping a focus on daily smoking/chewing, less than daily smoking/chewing or not at all. Other variables assessed were exposure of the study subjects in the past 30 days to second-hand smoke (at home/outdoor), quit attempts, anti-tobacco warnings on cigarette advertisements. Relevant independent variables included in the analysis were gender (male/female), age, place of residence, socioeconomic status where the education of head of the family, income per month, and occupation are considered, and the subject is categorized to be belonging to the upper class and lower class.

Statistical analysis

Data were entered in Microsoft Excel worksheet and analyzed using SPSS ver. 24.0. Descriptive statistics are presented in the form of frequency percentage. Chi-square test was used to study the association between the study variables.

## Results

Table 1 shows the distribution of study subjects by age, gender, and socioeconomic status. It was observed that there was a statistically significant difference observed between males and females. Approximately 2.4% of females smoked tobacco less than daily basis compared to 5.4% of males (*p* < 0.01).

**Table 1:** Distribution of study participants by age, gender, and socioeconomic status ^#^Fisher’s exact test *p < 0.05, statistically significant; *P *> 0.05, non-significant, NS

		Socioeconomic status		Total	Chi-square test	
		Upper class	Lower class		Chi-square value	p-value
Gender	Male	168	70	238	0.07	0.80 (NS)
		29.9%	29.0%	29.7%		
	Females	393	171	564		
		70.1%	71.0%	70.3%		
Age	18-20	501	207	708		0.54(NS)^#^
		89.3%	85.9%	88.3%		
	>20-24	60	34	94		
		10.7%	14 %	11.7%		

The association between tobacco use and gender is shown in Table 2. A statistically significant difference was observed with the smoking status between genders; 4.9% of the males smoked on a daily basis and 0.4% females smoked tobacco on daily basis (*p *< 0.05). Our study findings showed 5.4% of males and 2.4% occasional smokers who smoked less than daily. Approximately 58.5% of females among the current smokers were smoking on a daily basis in the past as compared to 45.5% males (*p *= 0.54). The current smokeless tobacco users in the study had 3% males and 1.5% females. Occasionally, tobacco was chewed on a less than daily basis by 2.6% males and 2.6% females (*p *= 0.36). It was also seen that there were no females in the study who chewed smokeless tobacco in the past or on a daily basis, whereas 40% of males chewed tobacco in the past and 1.8% chewed it on a daily basis.

**Table 2:** Measuring tobacco use prevalence (smoked/smokeless) in association with gender ^#^Fisher’s exact test *p < 0.05, statistically significant; *P *> 0.05, non-significant, NS

		Gender		Total	Chi-square test		
		Males	Females		Chi-square value		p-value
Current tobacco smoking status	Daily	11	2	13	24.17		<0.001*
		4.9%	0.4%	1.7%			
	Less than daily	12	13	25			
		5.4%	2.4%	3.3%			
	Not at all	200	520	720			
		89.7%	97.2%	95.0%			
Past daily smoking status	Yes	5	7	12	0.38		0.54(NS)
		45.5%	58.3%	52.2%			
	No	6	5	11			
		54.5%	41.7%	47.8%			
Past smoking status	Daily	5	2	7	-		0.003*^#^
		2.5%	0.4%	1.0%			
	Less than daily	7	6	13			
		3.5%	1.2%	1.8%			
	Not at all	186	504	690			
		93.9%	98.4%	97.2%			
Current smokeless tobacco use	Daily	7	8	15	2.05	0.36(NS)	
		3.0%	1.5%	1.9%			
	Less than daily	6	14	20			
		2.6%	2.6%	2.6%			
	Not at all	221	526	747			
		94.4%	96.0%	95.5%			
Past daily smokeless tobacco use	Yes	2	0	2	-	0.06(NS)^#^	
		40.0%	0.0%	10.5%			
	No	3	14	17			
		60.0%	100.0%	89.5%			
Past smokeless tobacco use	Daily	4	0	4	-	0.01*^#^	
		1.8%	0.0%	0.5%			
	Less than daily	5	9	14			
		2.3%	1.8%	1.9%			
	Not at all	210	500	710			
		95.9%	98.2%	97.5%			

Table 3 shows the association between socioeconomic status and current, past daily smoking status and past smoking status. 1.7% of the subjects from the upper and lower class were smoking on a daily basis whereas 3.4% from the upper class, 3.1% from the lower class smoked tobacco on a less than daily basis. No statistically significant difference was observed between the subjects from the upper and lower class (*p* = 0.97). The current non-smokers, subjects from upper class 1.2% smoked daily, 1% less than daily and lower class 0.5% smoked daily and 3.7% less than daily. The upper class is more likely to have smoked daily, less than daily as compared to the lower socioeconomic class (*p *= 0.04). It was observed that the prevalence of smokeless tobacco use on a daily basis was 1.8% among upper status and 2.1% of lower status and 2.4% in upper status and 3% in lower status on a less than daily basis which was not statistically significant (*p *= 0.84). Among less than daily smokeless tobacco users, 28.6% from lower status have used smokeless tobacco in the past, whereas none from the upper socioeconomic status have been used in the past.

**Table 3:** Measuring tobacco use prevalence (smoked/smokeless) in association with socioeconomic status ^#^Fisher’s exact test *p < 0.05 statistically significant; *p *> 0.05, non-significant, NS

		Socioeconomic status			Chi-square test	
		Upper class	Lower class		Chi-square value	p-value
Current tobacco smoking status	Daily	9	4	13	0.06	0.97(NS)
		1.7%	1.7%	1.7%		
	Less than daily	18	7	25		
		3.4%	3.1%	3.3%		
	Not at all	502	218	720		
		94.9%	95.2%	95.0%		
Past daily smoking status	Yes	11	1	12	-	0.07 (NS)^#^
		64.7%	16.7%	52.2%		
	No	6	5	11		
		35.3%	83.3%	47.8%		
Past smoking status	Yes	6	1	7	-	0.04*^#^
		1.2%	0.5%	1.0%		
	No	5	8	13		
		1.0%	3.7%	1.8%		
	Don’t know	483	207	690		
		97.8%	95.8%	97.2%		
Tobacco products smoked	Daily	10	3	13	-	0.71(NS)^ #^
		37.0%	27.3%	34.2%		
	Weekly	17	8	25		
		63.0%	72.7%	65.8%		
Current smokeless tobacco use	Daily	10	5	15	0.34	0.84(NS)
		1.8%	2.1%	1.9%		
	Less than daily	13	7	20		
		2.4%	3.0%	2.6%		
	Not at all	525	222	747		
		95.8%	94.9%	95.5%		
Past daily smokeless tobacco use	Yes	0	2	2	-	0.12(NS)^ #^
		0.0%	28.6%	10.5%		
	No	12	5	17		
		100.0%	71.4%	89.5%		
Past smokeless tobacco use	Daily	4	0	4	-	0.42(NS)^ #^
		0.8%	0.0%	0.5%		
	Less than daily	11	3	14		
		2.2%	1.4%	1.9%		
	Not at all	495	215	710		
		97.1%	98.6%	97.5%		

A summary description of the tobacco questions and the corresponding analysis indicators in association with the socioeconomic status of the subjects is shown in Table 4. No significant difference between the classes was observed when it came to frequency of subjects smoking inside their homes, on a daily, less than daily, monthly or less than a monthly basis (*p* = 0.91). It was observed that, among the subjects from the upper class, 48.1% attempted to quit smoking habit in the last 12 months when compared to subjects from lower status but the results were not statistically significant (*p *= 0.72). A statistically significant difference was observed between the classes when the overall sample was observed for noticing in the past 30 days (free sample of cigarettes, cigarettes at sale prices, coupons for cigarettes, free gifts or discounts on other products when buying cigarettes, clothing or other items with a cigarette brand name or logo, cigarette promotions in the mail (*p *< 0.05).

**Table 4:** Tobacco use questions and the corresponding analysis indicators in association with the socio-economic status of the subjects ^#^Fisher’s exact test *p < 0.05, statistically significant; *p* > 0.05 non-significant, NS

		Socioeconomic status			Chi-square test	
		Upper class	Lower class	Total	Chi-square value	P value
Frequency of smoking in the home	Daily	21	9	30	1.02	0.91(NS)
		3.9%	3.9%	3.9%		
	Weekly	20	6	26		
		3.8%	2.6%	3.4%		
	Monthly	6	3	9		
		1.1%	1.3%	1.2%		
	Less than monthly	7	2	9		
		1.3%	0.9%	1.2%		
	Never	478	212	690		
		89.8%	91.4%	90.3%		
Current working location	Yes	115	58	173	1.27	0.26(NS)
		20.5%	24.1%	21.6%		
	No	446	183	629		
		79.5%	75.9%	78.4%		
Currently working indoors or outdoors	Indoors	29	17	46	5.36	0.07(NS)
		25.2%	29.3%	26.6%		
	Outdoors	34	25	59		
		29.6%	43.1%	34.1%		
	Both	52	16	68		
		45.2%	27.6%	39.3%		
Smoking at the workplace	Yes	16	8	24	0.07	0.79(NS)
		21.9%	24.2%	22.6%		
	No	57	25	82		
		78.1%	75.8%	77.4%		
Attempting to quit smoking	Yes	13	4	17	-	0.72(NS)^ #^
		48.1%	36.4%	44.7%		
	No	14	7	21		
		51.9%	63.6%	55.3%		
Visiting a doctor	Yes	14	5	19	0.13	0.72(NS)
		51.9%	45.5%	50.0%		
	No	13	6	19		
		48.1%	54.5%	50.0%		
Receiving cessation advice from doctors	Yes	4	1	5	-	1.00(NS)^ #^
		28.6%	20.0%	26.3%		
	No	10	4	14		
		71.4%	80.0%	73.7%		
Anti-cigarette information in newspapers/magazines	Yes	338	135	473	0.73	0.39(NS)
		68.8%	65.5%	67.9%		
	No	153	71	224		
		31.2%	34.5%	32.1%		
Noticing anti-cigarette information on television	Yes	370	156	526	0.12	0.73(NS)
		73.1%	71.9%	72.8%		
	No	136	61	197		
		26.9%	28.1%	27.2%		
Noticing health warning on cigarette packs	Yes	334	146	480	0.18	0.91(NS)
		59.5%	60.6%	59.9%		
	No	150	61	211		
		26.7%	25.3%	26.3%		
	Did not see	77	34	111		
		13.7%	14.1%	13.8%		
Thinking about quitting because of warning on cigarette packs	Yes	156	60	216	0.11	0.75(NS)
		37.6%	36.1%	37.2%		
	No	259	106	365		
		62.4%	63.9%	62.8%		
Cigarette advertising in store	Yes	153	70	223	0.82	0.37(NS)
		31.6%	35.2%	32.7%		
	No	331	129	460		
		68.4%	64.8%	67.3%		
Noticing Cigarette promotions						
Free sample samples of cigarettes?	Yes	7	11	18	8.97	0.003*
		1.4%	5.2%	2.5%		
	No	498	199	697		
		98.6%	94.8%	97.5%		
Cigarettes at sale prices?	Yes	16	10	26	0.96	0.33(NS)
		3.2%	4.7%	3.6%		
	No	485	203	688		
		96.8%	95.3%	96.4%		
Coupons for cigarettes?	Yes	2	5	7	-	0.03*^#^
		0.4%	2.4%	1.0%		
	No	502	207	709		
		99.6%	97.6%	99.0%		
Free gifts /discounts on cigarettes?	Yes	2	5	7	-	0.03*^#^
		0.4%	2.4%	1.0%		
	No	498	206	704		
		99.6%	97.6%	99.0%		
Clothing or items with cigarette brand name or logo?	Yes	6	4	10	-	0.49(NS)^ #^
		1.2%	1.9%	1.4%		
	No	493	207	700		
		98.8%	98.1%	98.6%		
Cigarette promotion in the mail?	Yes	3	6	9	-	0.02*^#^
		0.6%	2.8%	1.3%		
	No	496	206	702		
		99.4%	97.2%	98.7%		

## Discussion

The present study reports information about tobacco use among students belonging to the upper and lower socioeconomic strata. Smoking has a bigger magnitude among men than smokeless tobacco. However, both are consistent as the prevalence is considerably more among the males belonging to the upper socioeconomic status. The result of the use of smoked tobacco being more can be attributed to the addictive nature of tobacco consistent with other findings [14]. Low prevalence of tobacco use among the study population was observed. This could be suggestive of the present ban on advertising, and Cigarettes and other Tobacco Products Act (COTPA) in India have been effective tools in curbing the tobacco menace.

The present study showed that there was no statistically significant difference between males and females and subjects belonging to the upper and lower class regarding smoked and smokeless tobacco use. Interestingly, in this study, it was observed that females smoked less than daily more frequently than males. However, this pattern cannot be taken for granted or ignored. Research states that, worldwide, the prevalence of women smoking/ smokeless tobacco use is low, but the tobacco industry actively targets women, as an initiative to spend their market in using them to manufacture tobacco products like hand-rolled bidis, marketing, and also consumption as a symbol of women empowerment [15-18].

Socioeconomic differences and quit attempts in the present study did not reveal any significance but very few subjects from the low socioeconomic class tried to quit the smoking habit. E. Fernandez et al., T. Brown et al., and C.E. Sheffer et al. observed the socioeconomic differences in quit attempts, there could be a fiscal reason behind this quit attempt, in particular, tobacco taxation [19-20].

Our findings suggest that various cigarette promotional activities like free samples, cigarettes at the sale price, receiving coupons for purchase, gift, clothing, etc. were noticed by the participants in the last 30 days. The awareness of these specific types of cigarette promotional activities was high with a significant difference between the upper and lower class subjects. The study participants from the lower class more routinely noticed promotional activities by the tobacco companies; this could be attributed to the fact that these participants are the vulnerable targets for marketing, who are also less likely to support laws for smoke-free environments. The results of this study could be used to advocate the implementation of effective policies that show a higher impact among adults belonging to the lower socioeconomic class, such as raising prices of tobacco products and banning advertising, promotion, and sponsorship of tobacco products [21-22].

A potential limitation of our study is the possibility of the recall bias during the interview on tobacco use. A possibility of underestimation of the tobacco use by the respondents is expected as the subjects may hide the truth.

## Conclusions

Our study results can conclude that the tobacco use pattern among various socioeconomic groups is insignificant, future attempts to reduce the tobacco use may not be specific for subgroups of the population. Meanwhile, efforts should be made to focus on people belonging to low socioeconomic strata, them being the easy targets for tobacco promotional activities by the tobacco industry. Still, considerable potential for development and implementation of effective strategies aimed at reducing tobacco use among different socioeconomic groups is the need of the hour.
